# Disseminated *Ureaplasma urealyticum* Infection and Hyperammonemic Encephalopathy in a Patient With Activated PI3K Delta Syndrome 2

**DOI:** 10.1093/ofid/ofaf084

**Published:** 2025-02-17

**Authors:** Yi Wye Lai, Ray Junhao Lin, Matthias Maiwald, Gareth Zigui Lim, Pooja Rao, Tse Hsien Koh, Ser Hon Puah, Teck Choon Tan, Hwee Siew Howe, Xin Rong Lim

**Affiliations:** Department of Rheumatology, Allergy and Immunology, Tan Tock Seng Hospital, Singapore; Department of Infectious Diseases, Woodlands Health, Singapore; Department of Pathology and Laboratory Medicine, KK Women's and Children's Hospital, Singapore; Department of Microbiology and Immunology, Yong Loo Lin School of Medicine, National University of Singapore, Singapore; Pathology Academic Clinical Programme, Duke–National University of Singapore Graduate School of Medicine, Singapore; National Neuroscience Institute, Department of Neurology, Tan Tock Seng Hospital, Singapore; Department of Laboratory Medicine, Tan Tock Seng Hospital, Singapore; Department of Microbiology, Singapore General Hospital, Singapore; Department of Respiratory and Critical Care Medicine, Tan Tock Seng Hospital, Singapore; Lee Kong Chian School of Medicine, Nanyang Technology University, Singapore; Department of Rheumatology, Khoo Teck Puat Hospital, Singapore; Department of Rheumatology, Allergy and Immunology, Tan Tock Seng Hospital, Singapore; Department of Rheumatology, Allergy and Immunology, Tan Tock Seng Hospital, Singapore

**Keywords:** hyperammonemia encephalopathy, hyperammonemia syndrome, *Ureaplasma* infection, *Ureaplasma urealyticum*

## Abstract

Hyperammonemia syndrome (HS) from *Ureaplasma* infection is typically reported in posttransplant recipients, particularly lung transplant. We describe a young woman with activated PI3K delta syndrome 2 who presented with HS from disseminated *Ureaplasma urealyticum* infection with septic arthritis. We also performed a literature review of *Ureaplasma*-associated HS in nontransplant patients.

## CASE REPORT

A 31-year-old Chinese woman with activated PI3K delta syndrome (APDS) 2 presented in June 2024 with 4 days of progressive polyarthritis affecting the wrists, elbows, knees, and ankles.

In 2017, at 24 years of age, she was diagnosed with PIK3R1 mutation (c.1425 + 1G > A) and had recurrent sinopulmonary infections, herpes zoster, maxillary sinus fungal mycetoma, bronchiectasis, hypogammaglobulinemia, chronic lymphoproliferation (persistent lymphadenopathy, development of mucosa-associated lymphoid tissue [MALT] lymphoma of the parotid glands in 2014, with recurrence in 2017), and autoimmunity. She displayed signs of systemic lupus erythematosus and Sjögren syndrome such as a malar rash, oral ulcers, Raynaud phenomenon, keratoconjunctivitis, arthralgia, cutaneous vasculitis, hypocomplementemia, positive anti-double-stranded DNA, and anti-Ro antibodies. She was on monthly replacement intravenous immunoglobulin (IVIG) and prednisolone at doses of 5–10 mg per day and had previously been treated with azathioprine, sirolimus, and multiple courses of rituximab.

She developed arthritis of the right elbow in June 2023, followed by arthritis of the right midfoot in December 2023 and right knee in April 2024. Repeated arthrocentesis of the right elbow joint was negative on Gram stain examinations and aerobic, anaerobic, mycobacterial, and fungal cultures. From November 2023 to April 2024, she missed medical appointments and treatment, including IVIG replacement. She was sexually active with her male partner and developed per-vaginal discharge in September 2023, which was treated with an antifungal vaginal pessary. In early 2024, she was diagnosed and treated for bacterial vaginosis in another facility. She was admitted for right corneal melt and arthritis of the right elbow in April 2024. As the ophthalmologist opined that the corneal melt was related to corticosteroid use, cultures from the right cornea were not obtained, and treatment involved application of corneal glue. Debridement and washout of the right knee and elbow under anesthesia was performed; no pus was seen intraoperatively. Chronic inflammation was found on synovial histology; stains for fungi and acid-fast bacteria were negative. Empiric treatment for septic arthritis with intravenous (IV) ceftriaxone and oral doxycycline therapy was stopped after a week, as synovial fluid cultures for bacteria, fungi, and mycobacteria, as well as polymerase chain reaction (PCR) tests for gonococcus and chlamydia, were all negative. As all microbiology was negative, she was treated for inflammatory arthritis with IV rituximab 1 g for 2 doses and prednisolone up to 20 mg/day and was reinstituted on monthly replacement IVIG. She responded clinically to treatment; however, approximately a month later, her symptoms recurred, prompting another admission in June 2024.

On examination, she was afebrile, with normal vital signs. There were multiple tender and swollen elbow, wrist, hand, knee, and ankle joints. Cardiovascular, respiratory, abdominal, and integumentary examinations were normal. Investigations revealed anemia with hemoglobin (Hb) 7.7 (11.8–14.6) g/dL, leukocytosis with white blood cell (WBC) count 14.7 (4.0–9.6) × 10^9^ cells/L, reactive thrombocytosis with platelet count 409 (15–360) × 10^9^/L, and C-reactive protein (CRP) of 299.3 (0.0–5.0) mg/L. Magnetic resonance imaging (MRI) of her right ankle showed tenosynovitis of the extensor tendons anterior to the ankle joint, with no evidence of abscess or osteomyelitis.

On day 5 of admission, she developed a single febrile episode of 38.6°C without any localizing symptoms. Blood cultures were negative and computed tomographic scan of the abdomen and pelvis revealed no occult source of infection. Urine cultures grew extended-spectrum β-lactamase–producing *Klebsiella pneumoniae* and *Escherichia coli*, which were treated with a 10-day course of ertapenem.

She was also diagnosed with major depressive disorder during her admission. She refused proposed key diagnostic investigations such as repeat arthrocentesis and washout, orthopedic consultation, and treatment, including antidepressants.

**Figure 1.**
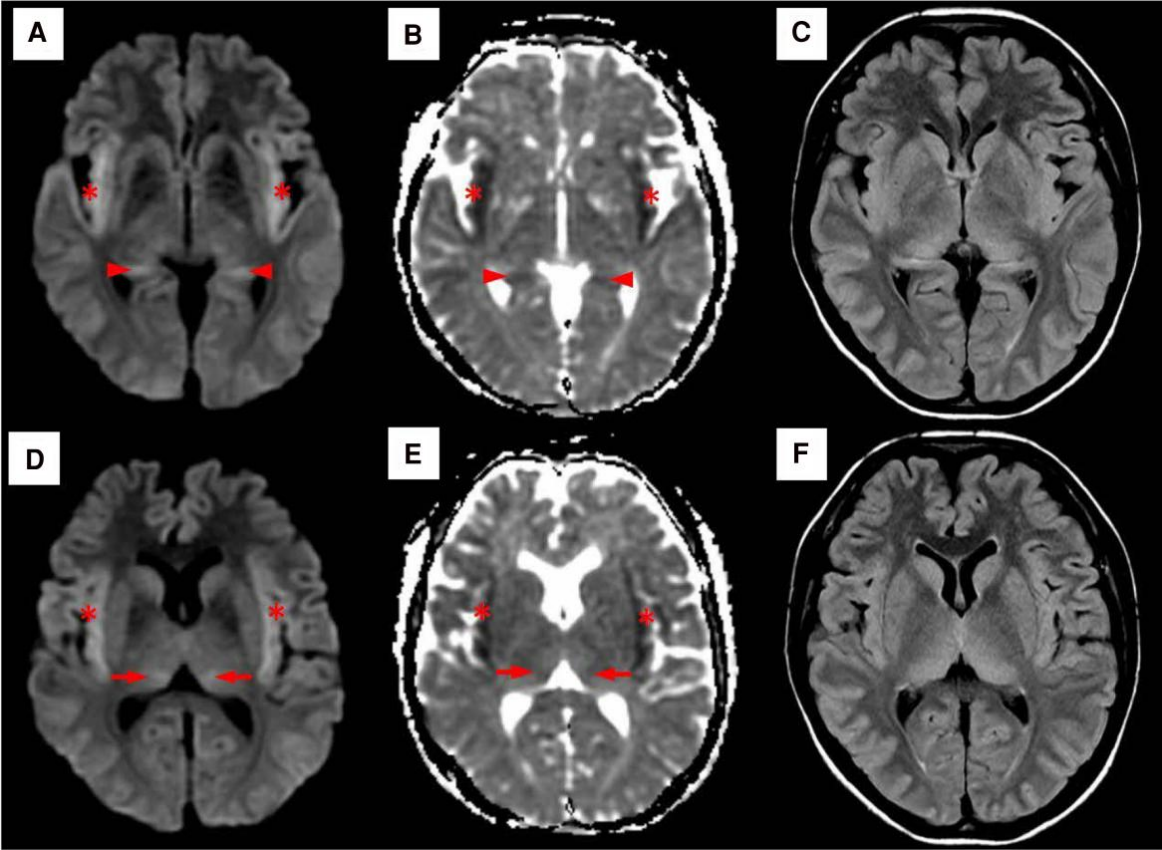
Axial brain magnetic resonance imaging showed bilateral symmetrical diffusion-weighted imaging hyperintense (*A* and *D*) and apparent diffusion coefficient hypointense (*B* and *E*) signal abnormalities in the bilateral insula (*), medial thalami (arrows), and hippocampi (arrowheads), consistent with restricted diffusion in these areas. These regions also showed corresponding symmetrical T2 fluid-attenuated inversion recovery hyperintensities (*C* and *F*).

**Figure 2.**
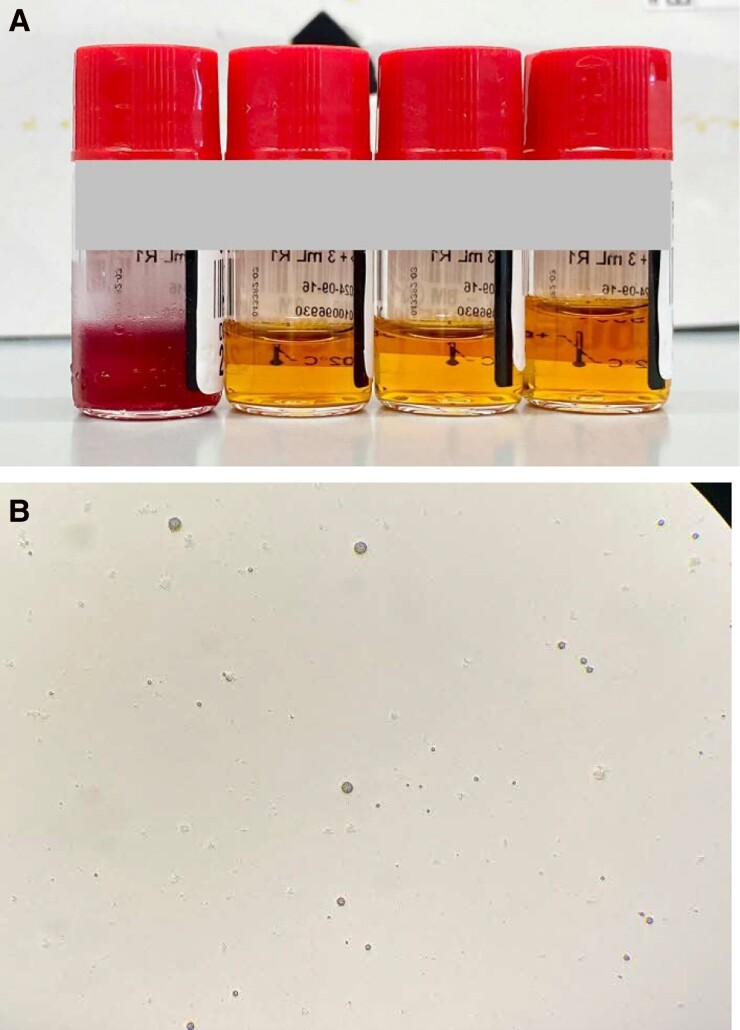
*A*, R2 medium from Urée-Arginine LYO 2 kit (bioMérieux SA, Marcy-l’Etoile, France). From left, uninoculated bottle and inoculated bottles at neat, 1:10, and 1:100 showing a color change indicating hydrolysis of urea. *B*, Small dark colonies of variable size seen on A7 agar (ELITech Microbio, Signes, France).

On day 29 of admission, she was found unarousable (Glasgow Coma Scale E1V1M1) with right gaze deviation and was intubated and transferred to the intensive care unit. Investigations showed anemia (Hb 9.6 g/dL), marked leukocytosis with neutrophilic predominance (WBC count 31.4 × 10^9^ cells/L), thrombocytosis (platelet count 800 × 10^9^/L), CRP 345.3 mg/L, marked hyperammonemia of >600 (16–52) μmol/L, hypogammaglobulinemia (immunoglobulin G, 4.3 g/L; immunoglobulin A, <0.4 g/L; immunoglobulin M, <0.3 g/L), with normal renal and liver function tests. Symmetrical areas of restricted diffusion and fluid-attenuated inversion recovery hyperintensities in bilateral insular regions and thalami (Figure 1) were seen on brain MRI, raising concerns for herpes simplex encephalitis. Lumbar puncture showed mild lymphocytic pleocytosis on cerebrospinal fluid (CSF) analysis (nucleated cells 11 [0–5] cells/μL, 85% lymphocytes) and negative results for all microbiological studies (aerobic, anaerobic, mycobacteria, and fungal culture; cryptococcus antigen; and PCR for herpes simplex virus, varicella zoster virus, cytomegalovirus, and *Toxoplasma gondii*). She subsequently developed status epilepticus (electroencephalography confirmed) that required 4 anti-epileptic agents for control. Bone marrow aspiration and biopsy was done due to previous history of MALT lymphoma and consideration of primary central nervous system (CNS) lymphoma and paraneoplastic encephalitis; this returned negative. She was empirically treated with IV acyclovir, ampicillin, ceftriaxone, and vancomycin for infective meningoencephalitis. She was also initiated on IV sodium benzoate and continuous renal replacement therapy (CRRT) for hyperammonemia, but CRRT was terminated when she developed hypotension and cardiac arrest. She eventually died on day 31 of admission. In a postmortem review, the clinical suspicion for *Ureaplasma*- or *Mycoplasma*-associated hyperammonemia prompted further investigations using her antemortem samples. Blood culture samples transferred to specialized medium grew *Ureaplasma* species (Figure 2). Five-plex PCR for *Trichomonas vaginalis*, *Mycoplasma genitalium*, *Mycoplasma hominis*, *Ureaplasma urealyticum*, and *Ureaplasma parvum* performed on residual bone marrow aspirate and synovial tissue from the knee obtained during surgical washout in April 2024 were also positive for *U urealyticum*, with all showing a cycle threshold value of approximately 32 (Figures 1 and 2).

## LITERATURE REVIEW

We searched PubMed and Embase from January 1990 to August 2024 for reports of hyperammonemia syndrome (HS) and *Ureaplasma* infection in nontransplant recipients (see details in the Supplementary material). We found 6 such cases; their clinical presentation, risk factors, and treatment are summarized in Table 1.

**Table 1.** Reported Cases of *Ureaplasma* Infections Presenting as Hyperammonemia Syndrome in Non–Transplant Recipients (Up through 2024)

First Author (Year)	Case Presentation	Microbiological Diagnosis	*Ureaplasma*: Directed Antimicrobials Received; Duration From Time of Presentation to Treatment Initiation	Clinical Outcomes	Risk Factors
Details included in table for reference Present Study (2024)	31-year-old woman with HS (altered mental status, status epilepticus, cerebral edema) and septic arthritis	Blood cultures, bone marrow aspirate, synovial tissue PCR returned positive for *Ureaplasma urealyticum*	Doxycycline (100 mg 12 h) for 1 wk given 2 mo prior to onset of HS, no *Ureaplasma*-directed antimicrobials given after onset of HS	Died	Primary immunodeficiency APDS, hypogammaglobulinemia, rituximab (1 g × 2 doses given 2 mo prior^a^) and low-to-moderate doses (5–20 mg/d) of prednisolone (started 1 y prior^a^)
Bharath (2023) [1]	57-year-old man with HS (status epilepticus, cerebral edema) and septic arthritis	Serum PCR positive for *Ureaplasma parvum*	Doxycycline^b^ for 8 d; 4 d	Died from complications of multiorgan failure, bowel ischemia	Dermatomyositis, seronegative rheumatoid arthritis, and interstitial lung disease on immunosuppression with methotrexate^b^, leflunomide^b^, prednisone^b^, mycophenolate mofetil^b^, and rituximab^b^
Rahman (2023) [2]	22-year-old woman with HS (encephalopathy, coma), pelvic abscess, serositis, and septic arthritis	Respiratory and urinary PCR positive for *U urealyticum*	Doxycycline^b^ for 2 d, then added-on azithromycin^b^ for another 5 d; not specified	Recovered after 7 d of antibiotics	Granulomatous polyangiitis on rituximab^b^ therapy; dose and onset of last dose not specified
Pan (2022) [3]	65-year-old man with HS (delirium, slurred speech, and agitation) and septic monoarthritis	Synovial fluid metagenomic sequencing test reported sequences of *U parvum*	NIL, discharged against medical advice	Died	Chronic hepatitis B, alcohol abuse, previous tuberculosis
Kok (2022) [4]	67-year-old woman with HS (altered mental status)	Blood culture positive for *U urealyticum*	Minocycline 100 mg q12h for 1 wk, then azithromycin 500 mg OD for 1 wk; 2 wk	Recovered but eventually died from other nosocomial infections	Pancreatic head adenocarcinoma, completed neoadjuvant chemotherapy 11 mo prior^a^ and adjuvant chemotherapy (tegafur, gemeraci, oteracil) given 3 mo prior^a^
Tawfik (2021) [5]	53-year-old woman with HS (status epilepticus, cerebral edema) and pneumonia	BAL PCR for *Ureaplasma*	Doxycycline^b^ for 4 d, then switched to levofloxacin^b^; 6 d	Withdrawal of care, died	Myeloablative allogeneic hematopoietic cell transplant 11 mo prior, CAR T-cell therapy (lympho-depleting therapy consisting of cyclophosphamide^b^ and fludarabine^b^) 2 wk prior complicated by subsequent cytokine release syndrome requiring 1 dose of tocilizumab^b^, IV 50 mg of methylprednisolone
Nowbakht (2019) [6]	32-year-old woman with HS (seizures)	Serum PCR panel positive for *U urealyticum*	Levofloxacin^b^; not specified	Died	B-cell ALL status post methotrexate, vincristine, pegasparaginase, and dexamethasone; diabetes mellitus (glycemic control not specified)

Abbreviations: ALL, acute lymphoblastic leukemia; APDS, activated PI3K delta syndrome; BAL, bronchoalveolar lavage; CAR, chimeric antigen receptor; HS, hyperammonemia syndrome; IV, intravenous; NIL, nothing; OD, once a day; PCR, polymerase chain reaction; q12h, every 12 hours.

^a^Time from initiation of chemotherapy/immunosuppression to onset of symptoms (if available).

^b^Doses of medications not specified.

## DISCUSSION

*Ureaplasma* species reside in the urogenital and respiratory tracts as part of the normal bacterial flora, but immunosuppression or instrumentation predisposes to penetration of the organism into the submucosa [7]. The unique characteristic of *Ureaplasma* species that metabolize urea for adenosine triphosphate production is typically inconsequential to the immunocompetent host, but when there is systemic infection, particularly in an immunocompromised host, this may pose a significant threat to ammonia clearance, leading to HS [8]. HS, characterized by excessive serum concentration of ammonia and resultant progressive neurologic dysfunction, is well-recognized in posttransplant patients [8]. There is a disproportionately increased incidence in lung transplant patients, in part contributed by donor respiratory tract colonization [8]. Our literature review of *Ureaplasma*-induced HS in nontransplant patients revealed risk factors of secondary humoral immunodeficiency, such as prolonged steroid use, rituximab use, B-cell acute lymphoblastic leukemia, myeloablation, chimeric antigen receptor T-cell therapy, malnutrition from chronic alcoholism, and chemotherapy [1–6]. In our patient, the primary site of infection appeared to be septic arthritis, probably arising from a urogenital source, with subsequent intensification of immunosuppression contributing to the development of HS.

Mortality is extremely high in nontransplant cases, even with appropriate treatment (∼83%, all cases died except 1), possibly contributed to in part by delays in diagnosis [1–6]. In comparison, the mortality of HS posttransplant is about 50%–70% [9].

*Ureaplasma* detection requires specialized PCR tests or media for culture, and infections respond poorly to commonly used empirical antibiotic regimens (eg, β-lactam antibiotics) [7, 10]. PCR assays for *Ureaplasma* performed on serum are not widely available and their performance characteristics are not well described. However, our review revealed several cases in which PCR on serum samples was used for diagnosis [1, 4, 6]. This seems to suggest that bacteremia is not uncommon in *Ureaplasma*-associated HS.

*Ureaplasma* is innately resistant to β-lactams, displaying susceptibility to mainly tetracyclines, macrolides, and fluoroquinolones [7]. We observed from our literature review that the 2 cases who recovered were treated with minocycline and doxycycline, as compared to the 2 cases treated with levofloxacin who died [2, 4–6]. This observation may be explained by naturally occurring mutations in the *parC* and *parE* regions of the *Ureaplasma* chromosome that confers resistance to fluroquinolones [11]. In the United States, the estimated prevalence of such mutations is 5%–6% [11].

Our patient continued to deteriorate despite 1 week of doxycycline treatment. Possible reasons for this may be due to the increased immunosuppression from treatment of possible reactive inflammatory arthritis, the bacteriostatic nature of doxycycline (compared to the bactericidal action of fluoroquinolones), and possibly an inadequate duration of treatment, considering that the literature review suggested that most cases of septic arthritis had treatment ranging several weeks up to months [12–15]. It may also be possible that the isolate had a high doxycycline minimum inhibitory concentration, but unfortunately antibiotic susceptibility testing was not available for us to test this hypothesis. Considerations of these aforementioned factors may influence the initial choice of antibiotics for empirical treatment of *Ureaplasma* infections and decisions for treatment duration.

In our patient, the diagnostic challenge was contributed to by the absence of fever; intermittent adherence to treatment, which hindered clinical assessment of treatment response; her refusal to cooperate with medical care and treatment; and the difficulty in differentiating infective arthritis from inflammatory arthritis. Existing literature on presentation of *Ureaplasma* septic arthritis in immunocompetent patients reports fever and suppurative arthritis [3, 15]. Conversely, immunocompromised patients may remain afebrile or only develop fever later on in the disease course, manifesting only with severe debilitating oligo-polyarthritis [1, 2, 14]. It is possible that our patient's underlying immunodeficiency with subsequent additional immunosuppression may have blunted her ability to mount a febrile response.

In retrospect, risk factors of severe hypogammaglobulinemia from underlying primary immunodeficiency, recent rituximab use, persistently raised CRP despite antibiotics, presence of HS without any liver pathology, and requirement of moderate to high doses of steroids for arthritis control should raise suspicion for an atypical infection. When her initial cultures returned negative, a referral to infectious diseases and extended microbial testing should have been considered.

MRI findings in HS typically include symmetrical signal abnormalities in the insula, cingulate cortices, and thalami [16]. In this case, the MRI brain findings were classic of hyperammonemic encephalopathy, necessitating urgent treatment with dialysis and ammonia scavengers (Figure 1) [16]. The presence of mild CSF pleocytosis may have suggested *Ureaplasma* CNS involvement; however, there were insufficient samples to test for this.

In summary, we present a case of fatal *U urealyticum* infection with acute and rapid deterioration, emphasizing the need for a heightened clinical suspicion in immunocompromised nontransplant hosts, in order to diagnose and institute management early. Although *Ureaplasma* infection manifesting as HS is typically associated with transplant recipients, it should also be considered in nontransplant immunocompromised patients with unexplained neurological dysfunction; more so in those with supportive neuro-radiological findings. Ammonia testing may be considered in this setting and, if elevated, should prompt PCR testing and incubation of microbiological samples in specialized culture media. Given that routinely used antibiotics are not active against *Ureaplasma* species, atypical antibiotic coverage should be added when the clinical suspicion is high.

## Supplementary Data

Supplementary materials are available at *Open Forum Infectious Diseases* online. Consisting of data provided by the authors to benefit the reader, the posted materials are not copyedited and are the sole responsibility of the authors, so questions or comments should be addressed to the corresponding author.

## Supplementary Material

ofaf084_Supplementary_Data

## Notes

***Patient consent.*** We confirm that our case report is exempted from institutional review board review. Details that might disclose the identity of the subject under study have been omitted, in accordance with Health Insurance Portability and Accountability Act standards. Consent was sought from the patient's next of kin as the patient is deceased.
