# Supplementary material for: Disseminated Ureaplasma urealyticum Infection and Hyperammonemic Encephalopathy in a Patient With Activated PI3K Delta Syndrome 2
Source: Open Forum Infect Dis. 2025 Feb 17;12(2):ofaf084. doi: 10.1093/ofid/ofaf084 (PMC11878574; doi:10.1093/ofid/ofaf084)
Supplement: ofaf084_Supplementary_Data [file ofaf084_supplementary_data.docx]

**Supplement information**

We conducted searches of the PubMed and Embase databases from January 1990 to Aug 2024. Using “hyperammonemia:ti,ab,kw AND ureaplasma:ti,ab,kw” as search terms on Embase, this yielded 83 results. Using ((ureaplasma[Title/Abstract]) OR (ureaplasma[MeSH Terms])) AND ((hyperammonemia[MeSH Terms]) OR (hyperammonemia[Title/Abstract])) Filters: from 1990 - 2024/8 as search terms on PubMed, this yielded 45 results. The references of retrieved records were also screened for additional cases. Transplant-related and paediatric cases were excluded, whilst data from individual meta-analysis was extracted if they were *Ureaplasma*-induced, non-transplant and adult cases of HS[2]. The final selection yielded 6 case reports.
